# Protein Phosphatase 2A Mediates Dormancy of Glioblastoma Multiforme-Derived Tumor Stem-Like Cells during Hypoxia

**DOI:** 10.1371/journal.pone.0030059

**Published:** 2012-01-11

**Authors:** Christoph P. Hofstetter, Jan-Karl Burkhardt, Benjamin J. Shin, Demirkan B. Gürsel, Lynn Mubita, Ramana Gorrepati, Cameron Brennan, Eric C. Holland, John A. Boockvar

**Affiliations:** 1 Department of Neurological Surgery, Weill Cornell Brain Tumor Center, Weill Cornell Medical College, New York Presbyterian Hospital, New York, New York, United States of America; 2 Department of Neurosurgery and Department of Cancer Biology and Genetics, Memorial Sloan-Kettering Cancer Center, New York, New York, United States of America; The University of Chicago, United States of America

## Abstract

**Purpose:**

The hypoxic microenvironment of glioblastoma multiforme (GBM) is thought to increase resistance to cancer therapies. Recent evidence suggests that hypoxia induces protein phosphatase 2A (PP2A), a regulator of cell cycle and cell death. The effects of PP2A on GBM tumor cell proliferation and survival during hypoxic conditions have not been studied.

**Experimental Design:**

Expression of PP2A subunits and HIF-α proteins was measured in 65 high-grade astrocytoma and 18 non-neoplastic surgical brain specimens by western blotting. PP2A activity was measured by an immunoprecipitation assay. For *in vitro* experiments, GBM-derived tumor stem cell-like cells (TSCs) were exposed to severe hypoxia produced by either CoCl_2_ or 1% O_2_. PP2A activity was inhibited either by okadaic acid or by shRNA depletion of the PP2A C subunit. Effects of PP2A activity on cell cycle progression and cell survival during hypoxic conditions were assessed using flow cytometry.

**Results:**

In our patient cohort, PP2A activity was positively correlated with HIF-1∝ protein expression (P = 0.002). Patients with PP2A activity levels above 160 pMP had significantly worse survival compared to patients with levels below this threshold (P = 0.002). PP2A activity was an independent predictor of survival on multivariable analysis (P = 0.009). In our *in vitro* experiments, we confirmed that severe hypoxia induces PP2A activity in TSCs 6 hours after onset of exposure. PP2A activity mediated G1/S phase growth inhibition and reduced cellular ATP consumption in hypoxic TSCs. Conversely, inhibition of PP2A activity led to increased cell proliferation, exhaustion of intracellular ATP, and accelerated P53-independent cell death of hypoxic TSCs.

**Conclusions:**

Our results suggest that PP2A activity predicts poor survival in GBM. PP2A appears to reduce the metabolic demand of hypoxic TSCs and enhances tumor cell survival. Modulation of PP2A may be a potential target for cancer therapy.

## Introduction

Glioblastoma multiforme (GBM) account for approximately 70% of all malignant astrocytomas and leave patients with a median survival of 15 months despite aggressive therapies including surgical resection, radiation, and chemotherapy [1]. Pathological characteristics of GBM include hypercellularity, vascular endothelial proliferation, and foci of necrosis surrounded by pseudopalisades [2]. The imbalance between rapidly proliferating cells, which are driven by RTK/RAS/PI3K signaling [3], [4], [5], and a poorly organized vasculature gives rise to a severely hypoxic microenvironment in GBM [6]. Severe cellular hypoxia in GBM is further aggravated by intravascular thrombosis due to up-regulation of tissue factor [7]. Ultimately, this leads to areas of necrosis surrounded by densely packed hypoxic astrocytoma cells, which are less proliferative and show a higher level of apoptosis compared to adjacent cells [8]. Pseudopalisades with severely hypoxic conditions have been proposed to select for more malignant and invasive neoplastic cells. Moreover, the slowly cycling nature of hypoxic tumor cells makes them more resistant to chemotherapy and irradiation [9]. Understanding the mechanisms that mediate tumor cell dormancy in response to oxygen deprivation could provide potential targets for therapeutic intervention.

Recent studies have shown that hypoxia induces enzymatic activity of protein phosphatase 2A (PP2A) in both *in vivo* [10] and *in vitro* [11], [12] models. PP2A is a heterotrimer consisting of an active core dimer, composed of the catalytic subunit (C subunit) and a scaffold protein (A subunit), which is joined by a regulatory subunit (B subunit) that dictates substrate specificity and enzyme activity [13]. A multitude of families, isoforms, and splice variants of the B subunit allow for the generation of more than 60 different heterotrimeric PP2A holoenzymes [14]. The complexity of the PP2A composition provides the molecular basis necessary for regulation of many cellular processes including proliferation, malignant transformation, differentiation, and apoptosis. The involvement of PP2A in regulation of cell proliferation was initially discovered when okadaic acid (OA), a PP2A inhibitor [15], was found to promote tumor growth in skin [16], stomach, and liver cancer models [17]. Inhibition of PP2A by various agents such as OA, SV 40, small tumor antigen, or PME has been shown to augment cellular proliferation in part by activation of RTK/RAS/PI3K signaling [18], [19]. PP2A also regulates cell proliferation by controlling cell cycle progression. Induction of PP2A activity by ceramide inhibits cell growth and leads to G1/S cell cycle arrest [20]. A previous study proposed that PP2A may mediate its effects on cell cycle progression as a physical complex with cyclin G2 [21]. Cyclin G2 is an unconventional cyclin that, independent of P53, causes cell cycle arrest or apoptosis [22]. Glioma and other cell lines up-regulate cyclin G2 in response to hypoxic conditions [23], [24], [25]. However, the role of PP2A signaling and cell cycle arrest in hypoxic tumor stem cells remains unclear.

In the current study, we aimed to investigate PP2A protein expression and activity in GBM. Moreover, we attempted to study whether hypoxia induces PP2A activity in GBM and whether PP2A activity is involved in regulation of cell cycle progression and survival of severely hypoxic tumor cells.

## Methods

### Patient cohort and surgical tissue samples

A total of 65 tumor samples were analyzed for this study. According to World Heath Organization (WHO) criteria, 62 samples were histologically characterized as grade IV glioblastoma multiforme (GBM) and three samples as grade III astrocytoma [2]. In five patients, tumor samples were collected from the initial tumor resection and again during resection of recurrent tumor. Minimum one-year clinical follow-up data was available on 54 GBM patients. Non-neoplastic brain tissue samples were collected from 18 patients who underwent temporal lobectomy for medically intractable seizures. All surgical tissue specimens were reviewed by our attending neuropathologist to ensure that each specimen was histologically consistent with either high grade astrocytoma or control tissue without evidence of neoplasia. Control tissue, however was not entirely normal neocortex. Pathological review detected neuronal dysplasia and gliosis in 6 specimens and focal neuronal damage in 4 of our 18 control specimens. Tumor volume was estimated by an ellipsoid model using the product of the maximal anteroposterior, lateral, and rostrocaudal radius measured on preoperative contrast-enhanced MR studies [26]. This study was approved by the Weill Cornell Medical College Institutional Review Board. Written informed consent was obtained from all patients prior to inclusion in the current study.

### Genomic data from the Cancer Genome Atlas

PP2A-C gene expression data of the Cancer Genome Atlas [3] was analyzed using the cBIO Cancer Genomics Portal (http://www.cbioportal.org). Analysis was carried out in 197 primary GBM following exclusion of secondary GBMs harboring IDH mutations [27], [28]. Z-scores were used to assess expression of mRNA.

### Generation of tumor stem-like cells

Operative tissue specimens were placed on ice immediately following harvest, mechanically dissected, and enzymatically dissociated at 37 C° for 30 minutes in PIPES/EDTA buffer containing papain (543 µgP/ml) and DNAse (8 U/ml). The resultant suspension was triturated using Pasteur pipettes to single cell suspension from cellular aggregates and filtered through a 70 µm nylon mesh. Enzymatic activity was quenched by adding 1 ml of fetal bovine serum followed by centrifugation for 5 minutes at 600 rpm. Supernatant was removed, and the pellet was resuspended and incubated in 10 ml of RBC lysis buffer (Invitrogen) for 10 minutes. After a second spin, cells were resuspended in stem cell medium consisting of DMEM/F12 containing B27 (Gibco), N2 (Gibco), 20 ng/ml EGF, basic-FGF (Invitrogen), L-glutamine (2 mM, Gibco), pyruvate (1 mM, Gibco), bovine serum albumin fraction V (0.06%, Gibco), and antibiotic/anti-mycotic (Gibco). They were then seeded at a density of 1×10^6^ cells per 10 cm cell culture dish (Nunc). Tumor stem-like cells (TSCs) were propagated as adherent cells in culture dishes coated with laminin (Sigma) at 10 µg/ml for 2 hrs prior to use [29]. The medium was refreshed every 48–72 hours. Prior to use in experiments, the tree TSCs used in the current study (334, 974 and 980) underwent a series routine tests confirming expression of stem cell markers, self-renewal, differentiation and tumorigenicity [30], [31] (Figure S1). TSCs were used for experiments between passage 5 and passage 20.

### Western blots

Tumor and non-neoplastic tissue samples were immediately placed on ice, dissected, and suspended in lysis buffer containing 2 mM EDTA, 0.15 M NaCl, and 10% glycerol. Following dissociation of the tissue using a glass grinder, extracts were collected by centrifugation at 14,000× g in a centrifuge at 4°C. Protein concentration was determined using a BCA protein assay kit (Thermo Scientific, IL, USA), and each lane was loaded with 35 µg of extract. Protein samples were separated by 4–12% SDS-PAGE and electrophoretically transferred to PVDF membranes. Membranes were subsequently blocked in 1× TBS-Tween20 with 5% BSA for 1 hr followed by incubation in primary antibody overnight at 4 C° under gentle agitation. Primary antibodies raised in rabbits against PP2A-B (PR 55, Cell signaling), P53 (Cell signaling), Phospho-P53 (S15, Cell signaling), β-actin (Cell signaling), HIF-2α (Abcam), and HIF-1α (Novus Biologicals), raised in mice against PP2A-C (BD Transduction Laboratories), HIF-1α (BD Transduction Laboratories), cyclin G2 (Abcam), and PME-1 (Santa Cruz Biotechnology), and raised in rat against PP2A-A (6F9, Covance) were used. Following three washes in TBS-Tween20, membranes were incubated in appropriate HRP-conjugated secondary antibody (Jackson ImmunoResearch) for 1 hr at room temperature. Immunoblotting was visualized by ECL detection solution (Thermo Scientific) for 5 minutes at room temperature.

### Immunoprecipitation

A total of 500 µg of protein extract was incubated with antibodies directed against PP2A-C (clone 1D6, Millipore) and Protein A agarose (30 µl) in 450 µl of RIPA buffer under constant agitation at 4° overnight. Following 3 washes with RIPA buffer, the sample was resuspended in electrophoresis sample buffer and processed as described above. The membrane was probed with antibodies directed against PP2A-C (6F9, Covance) and cyclin G2 (ProSci).

### PP2A activity assay

TSCs were plated at a density of approximately 20,000 cells per cm^2^ and cultured overnight. The next day, medium was replaced with fresh cell culture medium and cells were exposed to hypoxia either by using a hypoxic chamber (1% O_2_, BioSpherix) or by supplementing media with cobalt chloride (CoCl_2_) at a concentration of 200 µM. Cells were lysed at indicated time points using cell lysis buffer (Cell Signaling Technology) supplemented with protease inhibitor (Sigma-Aldrich). Lysates from each treatment group containing 100 µg protein were tested using a Malachite Green Phosphatase assay specific for serine/threonine phosphatase activity (Millipore). Measurements of PP2A activity in tumor samples and normal brain tissue samples were performed in the same conditions.

### Viral infections

TSCs were plated in 6-well plates at 50% confluency. The next day medium was changed to medium containing 5 µg/ml of polybrene and 20 µl of lentiviral particles encoding shRNA directed against random RNA (Control) PP2A-C∝, PME-1 or P53 (Santa Cruz Biotechnology). After 12 hours, the supernatant was removed and replaced with stem cell medium. One day later, cells were split 1∶3, grown for additional 2 days, and then selected with 2 µg/ml of puromycin. After 1 week in culture, puromycin dose was decreased to a maintenance concentration of 0.5 µg/ml.

### MTT and ATP assay

TSCs were plated in 96-well plates at a density of 20,000 cells per cm^2^. The following day media was replaced with fresh media. Hypoxia was induced by supplementing media with 200 µM of CoCl_2_ or by incubation in 1% hypoxia. At the same time OA was added for inhibition of PP2A as indicated. Viability and metabolic activity was assessed using an MTT kit according to the manufacturers instructions (Roche). ATP intracellular concentration was measured using the ViaLight® assay according to manufacturers instructions (Lonza). For measurement of the ATP decay following substrate deprivation, TSCs were incubated in D5030 media (Sigma) supplemented with 25 mmol/L HEPES without glucose.

### Flow cytometry

Cells were plated in 6-well plates at a density of 20,000 cells per cm^2^. After 24 hours, media was replaced with either normal growth media or media supplemented with 200 µM of CoCl_2_ and/or okadaic acid Following a 24-hour period, cells were exposed to a 1-hour pulse of 10 µM of BrdU, fixated, labeled with anti-BrdU antibody and total DNA was stained with 7-AAD (BrdU Flow Kit, BD Pharmingen). Following 1 week of hypoxia induced by either CoCl_2_ or 1% of hypoxia, apoptosis and cell death were assessed using Annexin V-FITC and Propidium Iodine (Trevigen). Fluorescence-activated cell sorting (FACS) analysis was carried out on 10,000 cells using a Becton-Dickinson FACScan (Becton Dickinson).

### Statistics

Continuous variables are presented as means ± standard error of mean. Categorical values are given as percentages. Correlations between continuous variables were calculated using Pearson's correlation analysis. Differences in continuous variables between two groups were assessed by a Mann-Whitney U-test and between three or more groups by ANOVA followed by Tukey post-hoc analysis. Kaplan-Meier survival analysis was performed for patients with high and low PP2A activity levels dichotomized at 160 pMP. Significant differences in survival were calculated using the log-rank test. Variables with significant prediction of survival in univariate analysis were entered into a multivariable analysis using the Cox's regression model. The model was built in a conditional forward stepwise fashion [32]. A P-value of less than 0.05 was considered significant. Statistical analyses were performed using SPSS (Version 19.0 for Macintosh).

## Results

### PP2A subunits exhibit a heterogenous expression pattern in surgical GBM specimen

**Figure 1:**
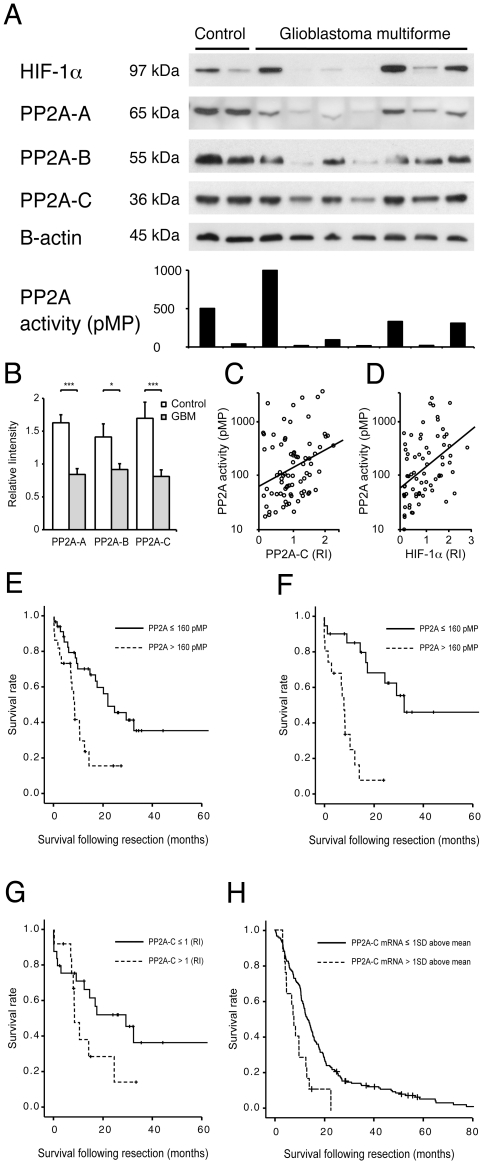
PP2A activity in GBM samples predicts poor survival. (A) Western blots of non-neoplastic brain tissue and GBM probed with antibodies directed against HIF-1α as well as against A, B and C PP2A subunits. The first control brain tissue sample exhibits neuronal dysplasia and gliosis on pathological review and expresses higher HIF-1α levels compared to the second control specimen with entirely normal histology. GBM specimens express variable levels of HIF-1α and PP2A subunits. Bar diagram represents PP2A activity of each sample analyzed in the western blot above. (B) Protein expression of all three PP2A subunits is significantly lower in GBM specimens (n = 65) compared to normal brain tissue (n = 18). (C) Protein expression levels of the PP2A C subunit are significantly correlated with PP2A activity (Pearson Correlation Coefficient 0.334, P = 0.003, Trend line R^2^ = 0.093). (D) HIF-1α protein expression levels correlate significantly with PP2A activity (Pearson Correlation Coefficient 0.36, P = 0.002, Trend line R^2^ = 0.184). (E) In our patient cohort, high PP2A activity is associated with poor prognosis. Kaplan Meyer analysis reveals significantly worse overall survival of patients with PP2A activity above 160 pMP (dotted line, n = 24) compared to patients with levels below 160 pMP (solid line, n = 36, P = 0.002). Thus median survival of patients with high PP2A activity is 8.1 months (confidence interval 6.6–9.6 months) compared to 21.0 months of patients with low PP2A activity (confidence interval 10.4–31.5 months). (F) The impact of PP2A activity on prognosis is more pronounced in patients at time of initial surgery. In this subgroup, median survival of patients with high PP2A activity is 7.7 months (n = 16, confidence interval 5.9–9.6 months) compared to 31.0 months of patients with low PP2A activity (n = 22, confidence interval 16.4–45.6 months, P<0.001). (G) At the time of initial surgery, patients with high PP2A-C protein expression (PP2A-C>1 relative intensity [RI]) exhibit a tendency towards worse overall survival (median survival 8.2 months, confidence interval 5.1–11.3 months) compared to patients with low expression (PP2A-C≤1 RI, median survival 28.1 months, confidence interval 11.1–43.8 months, P = 0.07). (H) High PP2A mRNA expression predicts poor survival in an independent dataset derived from the cancer genome atlas [3]. Patients with elevated PP2A-C mRNA expression (One standard deviation above the mean expression, dotted line, n = 17) had a significantly worse prognosis (P = 0.0014) compared to patients with lower expression levels (solid line, n = 179). In patients with high PP2A-C mRNA expression, the median survival was 7.7 months (confidence interval 4.7–13.0 months) compared to 13.6 months (confidence interval 11.9–15.4 months) in patients with low PP2A mRNA expression.

A total of 65 high-grade astrocytoma specimens from 60 patients were examined (Table 1). Patients were predominantly male (61.7%) and on average 56.7 years old. Altered mental status, seizures and/or headaches were the most common presenting symptoms. At the time of initial presentation, the mean Karnofsky score was 79.5. The average tumor volume was 43.9 cm^3^. Protein expression levels of all three PP2A subunits were analyzed in non-neoplastic brain tissue compared to GBM samples. Homogeneous expression levels of the A, B, and C subunits were detected in the 18 normal brain specimens. Consistent with a previous report [33], expression levels of the three subunits were decreased to variable degrees in the GBM specimens (Figure 1A and B). On average, PP2A expression was reduced by approximately one third in GBM compared to normal tissue samples (Figure 1A and B). There was a significant positive correlation between expression levels of the A and C subunits (Pearson Correlation Coefficient 0.76, P<0.001, Figure 1A).

**Table 1:** GBM Patient Characteristics (n = 60).

Age		56.7±12.5 yrs
Gender	Male	37 (61.7%)
	Female	23 (38.3%)
Karnofsky score		79.5±14.9
Main presenting symptom	Altered mental status	20 (33.3%)
	Seizure	15 (25.0%)
	Headache	11 (18.3%)
	Visual Impairment	7 (11.7%)
	Aphasia	5 (8.3%)
Tumor volume at presentation		43.9±34.9 cm^3^
Recurrent tumor	Yes	19 (31.7%)
	No	38 (63.3%)
	N/A	3 (5.0%)

### High PP2A activity is associated with poor prognosis in GBM patients

PP2A activity was measured in non-neoplastic brain specimens and in GBM samples. There was a tendency towards lower PP2A activity level in GBM specimens (344.1±76.6 pMP) compared to control brain tissue (448.7±166.7 pMP). However, this difference did not reach statistical significance due to great variability in both groups. PP2A activity followed a significant correlation with the protein expression of the C subunit (Pearson Correlation Coefficient 0.334, P = 0.003, Figure 1C). Patients with tumors exhibiting PP2A activity above 160 pMP had a significantly worse prognosis compare to patients with low PP2A activity (P = 0.002). Thus, patients with high PP2A activity had a median survival of 8.1 months (confidence interval 6.6–9.6 months) compared to patients with low PP2A activity who lived 21.0 months (confidence interval 10.4–31.5 months, Figure 1E). The impact of PP2A activity on overall survival was more pronounced in a subset of patients who underwent their initial surgical resection (Figure 1F). In this subgroup, median survival of individuals with high PP2A activity was 7.7 months (confidence interval 5.9–9.6 months) compared to 31.0 months of patients with low PP2A activity (confidence interval 16.4–45.6 months, P<0.001). In the same subset of patients, PP2A-C protein expression levels were associated with a tendency towards worse survival (Figure 1G). In order to account for known predictors of survival such as age and Karnofsky score [34] a multivariable analysis was performed. PP2A activity remained an independent predictor on survival (P = 0.009, Table 2). Importantly, independent validation of our results was obtained by analyzing mRNA expression data from the Cancer Genome Atlas (TCGA) [3]. Patients with elevated PP2A-C mRNA expression (one standard deviation above the mean expression) had a significantly worse prognosis (P = 0.0014) compared to patients with lower expression levels (Figure 1E). In patients with high PP2A-C mRNA expression, the median survival was 7.7 months (confidence interval 4.7–13.0 months) compared to 13.6 months (confidence interval 11.9–15.4 months) in patients with low PP2A mRNA expression.

**Table 2:** Cox multivariate analysis for survival prediction.

	Hazard ratio	SE	P-value	95% Confidence interval
Age	1.046	0.015	0.002	1.016–1.076
Karnofsky score	0.957	0.014	0.002	0.931–0.984
PP2A activity	1.001	0.000	0.009	1.000–1.001

### PP2A mediates G1/S phase growth inhibtion during severe hypoxia

In order to estimate the degree of hypoxia in our high-grade glioma specimens, we measured HIF-α protein expression. In the presence of oxygen, members of the alpha family of hypoxia-responsive proteins are continuously degraded and levels are low. During hypoxic conditions degradation is inhibited and the protein accumulates [35], [36]. Heterogeneous HIF-1α expression was detected in both control and GBM samples (Figure 1A). In non-neoplastic tissue higher HIF-1α expression was detected in specimens with neuronal dysplasia (n = 6) or focal neurological damage (n = 4) compared to control tissue with entirely normal histopathological appearance (n = 8, 1.6 relative intensity compared to 0.6 relative intensity, P = 0.002). In our cohort, HIF-1α expression was correlated with PP2A activity (Figure 1D, Pearson Correlation Coefficient 0.36, P = 0.002), while no correlation was detected between HIF-2α protein expression and PP2A-activity (Pearson correlation coefficient 0.027, P = 0.897).

**Figure 2:**
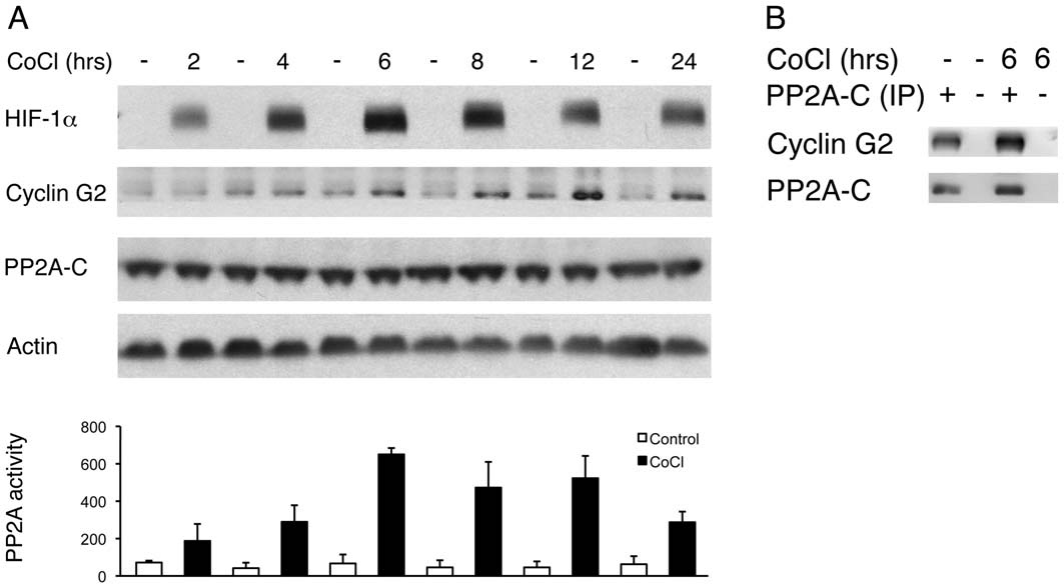
Hypoxia increases cyclin G2 expression and PP2A activity in TSCs. (A) At 2, 4, 6, 8, 12, and 24 hours following addition of CoCl_2_ to culture media, HIF-1α protein is detected in TSCs while cells grown in standard conditions lack expression. Increased cyclin G2 levels are detected in TSCs six hours following exposure to CoCl_2_. Expression levels of the PP2A C subunit remain stable throughout the experiment. Bar graph depicts marked increase of PP2A activity following exposure of TSCs to CoCl_2_. Thus, PP2A activity levels are nine times higher in TSCs propagated in CoCl_2_ for six hours compared to TSCs grown in standard conditions. Bars represent the mean value of 3 independent experiments ± SEM. (B) To determine whether PP2A directly interacts with cyclin G2, PP2A was immunoprecipitated in cell lysates from control TSCs and TSCs exposed to six hours of CoCl_2_. In both conditions, the PP2A C subunit forms a complex with cyclin G2.

We then studied the response of GBM-derived TSCs to hypoxia *in vitro*. Hypoxia led to increased HIF-α protein expression in TSCs (Figure 2A and Figure S2). Increased HIF-1α protein levels were consistently detected 2 hours following exposure to hypoxia while elevated HIF-2α protein levels were first noted at 72 hours. We then examined whether hypoxic conditions would induce PP2A activity of TSCs *in vitro*. First, we measured PP2A activity of TSCs grown in standard culturing conditions and found some baseline variation. For example, high PP2A activity was routinely detected in cultures that were highly confluent or had been propagated for several days without replating. Conversely, low PP2A activity was consistently observed in cell cultures that were plated at a density of 20,000 cells per cm^2^ within the previous 24 hours. Next, in order to test whether hypoxic conditions lead to increased PP2A activity *in vitro*, we exposed GBM-derived TSCs to hypoxia. While PP2A activity of TSCs grown at 80% confluency was low one day after plating, activity increased markedly from 6 hours onwards following exposure to CoCl_2_ (Figure 2A) or 1% hypoxia (Figure S2A). Given that cyclin G2, a binding partner of PP2A [22], has been shown to be upregulated in cancer cells in response to hypoxic conditions [23], [24], [25], we studied its temporal expression pattern in relation to PP2A activity following exposure to hypoxia. Six hours following supplementation of culture medium with CoCl_2_ and 12 hours following exposure to 1% hypoxia increased cyclin G2 expression levels were observed (Figure 2A, Figure S2). Elevated cyclin G2 expression levels mirrored the rise of PP2A activity. An immunoprecipitation assay confirmed that cyclin G2 formed a complex with PP2A-C in TSCs during both normoxic and hypoxic conditions (Figure 2B).

**Figure 3:**
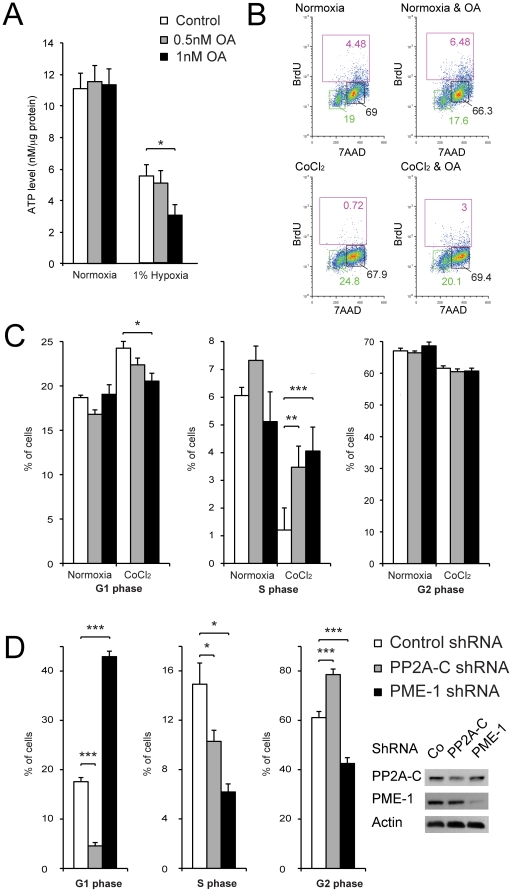
PP2A reduces ATP consumption and inhibits growth of TSCs during hypoxic conditions. (A) Effect of PP2A inhibition on ATP consumption of TSCs during normoxia and hypoxia. Intracellular ATP was measured in TSCs 1 hour following substrate depletion (D5030 media) with our without PP2A inhibition. PP2A inhibition leads to significantly higher ATP consumption during hypoxic conditions. The ATP content was measured and normalized to the lysate protein content. Bars represent the mean values of 8 independent experiments ± SEM. (B) 24 hours following plating, TSCs are exposed to CoCl_2_ and/or OA for additional 24 hours. Hypoxia leads to G1/S phase growth inhibition in TSCs. Representative examples of cell cycle analysis experiments. (C) Summary of six independent experiments reveals that hypoxia-mediated G1/S phase arrest is partially reversed by OA in a dose-dependent fashion. Accordingly, PP2A inhibition allows for significantly more hypoxic TSCs to progress into the S-phase. (D) In line with pharmacological inhibition of PP2A, depletion of PP2A by shRNA alleviates G1/S cell cycle arrest in TSCs propagated in CoCl_2_. Given the longer duration of PP2A inhibition in PP2A-depleted cells (48 h) compared to OA-treated cells (24 h [C]), more PP2A-depleted cells progress to G2 phase. Increasing PP2A activity by depletion of the endogenous PP2A inhibitor PME-1 has the opposite effect. Western blots reveal depletion of PP2A-C and PME-1 in TSCs by shRNA.

In order to explore the role of PP2A activity in hypoxic TSCs, we studied the effects of PP2A inhibition. During normoxic culturing conditions, inhibition of PP2A did not significantly alter ATP consumption rate of TSCs (Figure 3A). Conversely, during hypoxic conditions PP2A inhibition significantly increased ATP consumption of TSCs (Figure 3A). To further investigate the role of PP2A on the energy status of hypoxic TSCs we analyzed intracellular ATP levels at multiple time points following onset of hypoxia. PP2A inhibition significantly delayed adaptive reduction of metabolic activity as well as ATP production in hypoxic TSCs during the first 24 hours (Figure S3D). Later, increased metabolic rate/ATP demand by PP2A inhibition exhausted intracellular ATP levels. At 1 week following exposure, PP2A inhibition caused significant depletion of intracellular ATP in hypoxia TSCs. Similarly, PP2A inhibition led to decreased cell proliferation and viability of TSCs at 1 week following exposure to hypoxia (Figure S3B and C). To test whether exhaustion of intracellular ATP during PP2A-inhibtion of TSCs was caused by substrate deprivation, we repeated experiments in high glucose containing media. Supplementation of culture media with high concentration of glucose partially prevented exhaustion of ATP in TSCs grown for one week in in hypoxic conditions with concomitant PP2A inhibition (Figure S3E).

Cell cycle analysis revealed that TSCs exposed to hypoxic conditions for 24 hours exhibited G1/S phase growth inhibition (Figure 3B and C). While low doses of OA had only minimal effect on cell cycle progression of TSCs grown in standard culturing conditions, OA reversed the G1/S phase growth inhibition in a dose-dependent fashion in TSCs exposed to CoCl_2_ for 24 hours. In addition, a concomitant significant increase of TSCs progressing into S phase was observed. The role of PP2A on G1/S phase progression was further investigated in TSCs transduced with shRNA directed against random RNA (control), PP2A-C, or PME-1, which is an intrinsic antagonist of PP2A-C [19]. Depletion of PP2A-C promoted G1/S transition. Thus, three times fewer TSCs were found in the G1 phase following depletion of PP2A-C. By contrast, PME-1 depletion doubled the number of TSCs observed in the G1 phase (Figure 3D).

### PP2A activity increases cell survival during severe hypoxia

**Figure 4:**
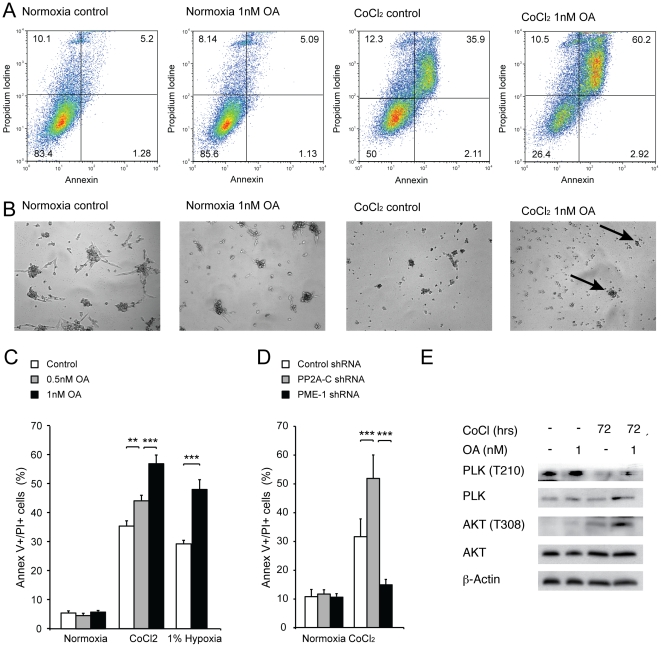
PP2A inhibition enhances cell death of hypoxic TSCs. (A) Examples of Annexin/PI stainings of TSCs grown for seven days in either normal culturing conditions or in media supplemented with 200 µM of CoCl_2_. Addition of 1 nM of OA has no significant effect on cells propagated in normal culturing conditions, but significantly increases the number of Annexin V+/PI+ cells in hypoxic conditions. (B) Photographs of TSCs *in vitro* following one week of culture during normoxia or hypoxia with or without inhibition of PP2A. Viable cells are stained by formazan dye. Few viable cells (black, arrows) are seen in hypoxic cells exposed to OA. (C) While neither 0.5 nM nor 1 nM of OA exerts a significant effect on the death of TSCs in routine culturing conditions, OA significantly increases the amount of Annexin V+/PI+ TSCs during hypoxic conditions. Bars represent the mean value of 6 independent experiments ± SEM. (D) Depletion of the PP2A-C causes an almost two-fold increase of cell death during hypoxic conditions. Importantly, enhancement of PP2A activity by PME-1 depletion reduces death of hypoxic TSCs to levels comparable to normoxia. (E) Inhibition of PP2A in TSCs exposed to hypoxic conditions for 3 days leads to increased phosphorylation of PLK and AKT.

While PP2A inhibition partially sustained cell proliferation during the first 24 hours of hypoxia, long-term PP2A inhibition eventually lead to decreased cell viability at one to two weeks (Figure S3B and C). In order to explore the mechanism underlying decreased viability caused by OA, TSCs grown in either normoxia or hypoxia for 1 week were stained with annexin/PI and subjected to flow cytometry. As expected, few dead TSCs were detected during normal culturing conditions (Figure 4A–C). Also, in line with our MTT results, exposure of TSCs to low doses of OA during normoxia had minimal effect on cell death. We then inhibited PP2A in TSCs propagated in hypoxic conditions for seven days. At that time, only a small amount of acute apoptosis was detected (1.9±0.3%, Annexin V +/PI −, Figure 4A). PP2A inhibition by 1 nM of OA led to a small increase of acute apoptosis during hypoxic conditions (3.1±0.3%, P<0.05). The vast majority of dead TSCs detected after 7 days of hypoxia were positive for Annexin V and PI, indicating that cell death was mainly due to late apoptosis (Figure 4A–C). Inhibition of PP2A gave rise to a massive increase of Annexin V+/PI+ cells during hypoxic conditions (Figure 4A–C). We then transduced TSCs with shRNA targeted against random RNA (control), PP2A-C, and PME-1. In annexin/PI cytometry experiments, depletion of PP2A-C had similar effects as OA. Thus, PP2A-C depleted TSCs exhibited significantly higher rates of cell death following culture in hypoxic conditions compared to culture in standard conditions (Figure 4D). Importantly, enhancement of PP2A signaling by depletion of PME-1 as previously described [19] decreased levels of cell death in hypoxic conditions to levels comparable to normoxic conditions. In line with previous reports [37], [38], we detected increased levels of posphorylated PLK and AKT in hypoxic cells treated with OA (Figure 4E). We then investigated the role of P53 in cell death of hypoxic TSCs during PP2A inhibition (Figure S4). Severe hypoxia led to dephosphorylation of P53 at the serine15 residue and reduction of P53 expression in TSCs. Treatment with OA partially restored P53 expression in hypoxic TSCs. In order to investigate whether P53 mediates TSC death during hypoxia with concurrent PP2A inhibition, we depleted P53 in TSCs using shRNA. Flow cytometry revealed that P53 depletion did not alter the amount of cell death during hypoxic culture conditions with concurrent PP2A inhibition.

## Discussion

In the current study, we propose that increased PP2A activity predicts poor outcome in patients with GBM. *In vitro* experiments suggest that hypoxia induces PP2A activity in GBM-derived TSCs. Hypoxia-induced PP2A decreases metabolic activity by halting cell proliferation and enhances survival of TSCs in a severely hypoxic environment.

Severely hypoxic GBM with high PP2A activity carried a poor prognosis in our patient cohort. Several studies have linked severity of hypoxia in malignant astrocytoma with a more aggressive clinical course [6], [39]. Evans and colleagues measured tissue hypoxia using the 2-nitroimidazol agent EF5 and demonstrated modest cellular hypoxia in low-grade astrocytomas (WHO grade 2) and severe levels of hypoxia in GBMs (WHO grade 4) [6]. Thus, severity of hypoxia was correlated with histological tumor grade in malignant astrocytomas [6], [39]. However, when accounting for tumor grade, HIF-1∝ was no longer an independent predictor of survival [39]. This is similar to findings in our series. While HIF-1∝ was a predictor of survival performing a univariate analysis, statistical significance was lost on multivariable analysis (data not shown). Given the strong association between HIF-1α and PP2A, one may question why PP2A activity is an independent predictor of survival. It may be speculated that PP2A-mediated dormancy of TSCs protects tumor cells from a variety of other toxic factors such as deprivation of nutrition or exposure to chemotherapy or irradiation.

Brain tumors have high glucose consumption since tumor cells commonly use inefficient glycolysis for ATP production [40], [41], [42]. Thus, decreased glucose concentration is a common finding in centers of solid brain tumors [43], [44]. Glucose deprivation combined with hypoxia results in cell death of malignant glioma cell lines with phenotypic features of late apoptosis/necrosis [45]. We similarly showed that the major phenotype of cell death in hypoxic TSCs during PP2A inhibition was late apoptosis/necrosis. Conversely, acute apoptosis was almost absent. Interestingly, a recent study demonstrated a dramatic increase of hypoxia-induced cell death in HIF-1∝-depleted ovarian cancer cells [46]. Similar to PP2A inhibition, HIF-1∝ depletion prevented cancer cells from arresting in the G1/S phase during hypoxic conditions. HIF-1∝- depleted cancer cells continued to proliferate, depleted intracellular ATP levels, and eventually acceleration of late apoptosis/necrosis. Cell death may have been caused by the lack of mitochondrial shut-down during severe hypoxia leading to production of reactive oxygen species and concomitant cell death.

There is also a body of literature suggesting that PP2A plays a critical role in activation of DNA damage checkpoints, which leads to arrest of cell cycle progression and allows for DNA repair [47], [48], [49], [50]. Specifically, PP2A has been shown to mediate the DNA damage checkpoint responses by activating ATR and Chk1 kinases following γ-irradiation [51]. Moreover, DNA damage by chemotherapy or radiation has been proposed to activate the DNA damage checkpoint via PP2A-mediated dephosporylation of PLK [52]. PP2A inhibition leads to phosophorylation and activation of PLK and allows for enhanced anticancer activity of chemotherapy directed against a GBM cell line [37] as well as a pheochromocytoma cell line [38]. Thus, PP2A might promote tumor cell survival via halting cell cycle progression in response to a variety of cell-toxic environmental factors.

In our experiments, we found that severe hypoxia (1% O_2_) or CoCl_2_ induces PP2A activity in TSCs *in vitro*. Increased PP2A activity in response to hypoxia has been previously reported in a model of transient cerebral ischemia with reperfusion *in vivo* [10]. Similarly, hypoxic culture conditions give rise to increased PP2A activity in cortical primary astrocytes *in vitro* [11]. The detailed composition of the hypoxia-induced PP2A complex and possible mechanisms of PP2A activation remain area of intensive research. We speculate that hypoxia-induced PP2A forms a complex with cyclin G2 to affect cell cycle progression. In line with previous studies, we found that cyclin G2 was upregulated during hypoxic conditions [23], [24], [53] and increased cyclin G2 protein levels mirrored the increase in PP2A activity. Cyclin G2 is an unconventional cyclin that leads to G1/S cell cycle arrest by inhibition of CDK2 activity independent of P53 [21]. Importantly, cyclin G2 forms a complex with PP2A, and its phosphatase activity is suppressed by low doses of okadaic Acid (OA, <2 nM) [21]. Moreover, in line with observations by Bennin and colleagues [21], low doses of OA (1 nM) were sufficient to partially reverse G1/S cell cycle arrest in hypoxic cells. Thus, we suggest that PP2A contributes to the G1/S phase arrest in combination with cyclin G2 during hypoxic conditions. In our *in vitro* experiments, we observed adaptive changes of RTK/RAS/PI3K signaling in TSCs in response to hypoxia consistent with previous literature [54], [55], [56]. Regulation of RTK/RAS/PI3K signaling aims to regulate metabolic functions and proliferation in order to optimize survival under hypoxic conditions. PP2A has been shown to interact with RTK/RAS/PI3K signaling at multiple levels and is mainly opposing its effects (for review, please see [57]). One may speculate that the necrotic-like cell death observed in our model following PP2A inhibition may have resulted from increased AKT activity and subsequent enhancement of metabolic activity and cell proliferation in a non-growth-permissive hypoxic environment. PP2A inhibition has been proposed to increase tumor cell death during chemotherapy via a similar mechanism [37], [38]. Moreover, inhibition of RTK/RAS/PI3K signaling has been suggested to increase cell survival during hypoxic conditions [58]. Thus, Satoh and colleagues found that a MAPK/ERK kinase inhibitor (U0126) promoted survival of primary cortical cultures during severe hypoxia (O_2_<0.2%).

While we demonstrate the role of PP2A in adaptive cellular response to hypoxia, several areas require further investigation prior to the design of PP2A-based experimental therapies. First, the composition of hypoxia-induced PP2A heterotrimers and the mechanism of their activation [12], [59] are poorly understood. Moreover, since PP2A activity is involved in numerous cellular processes, systemic PP2A inhibition would also affect PP2A signaling in normoxic tissues, possibly causing adverse effects. This is of particular importance since the therapeutic range of pharmacological PP2A inhibition targeting hypoxic cells is narrow. Moreover, OA is not a specific inhibitor for PP2A [60]. While OA doses used in the current study are similar to the IC_50_ dose for PP2A inhibition (0.1 nM) [15], OA at higher concentrations (IC50 = 10 nM) also blocks PP1. However, results from initial animal experiments using LB1.2 for systemic PP2A-inhibition in combination with chemotherapy are encouraging. PP2A-inhibition increased the anticancer effect of temazolomide in xenograft mouse model for GBM and neuroblastoma [37]. Systemic PP2A-inhibition was tolerated in rodents, and no adverse effects were reported during a short follow-up period [37], [38].

In conclusion, increased PP2A activity is detected in hypoxic GBM specimens of patients carrying the worst prognosis. PP2A mediates reduction of energy consumption of hypoxic TSCs, which enhances tumor cell survival. Future studies need to address whether increased PP2A activity also contributes to the increased therapy resistance of hypoxic tumor cells [9] and to further examine possible synergistic effects of chemotherapy with PP2A inhibition [37], [38]. Hypoxia-induced PP2A activity promotes survival of TSCs and may be a possible target for experimental cytotoxic therapies.

## Supporting Information

Figure S1 Single cell clonal analysis for stemness and differentiation. Dissociated GBM tissue was seeded on laminin coated 10 cm dishes (3×10^6^ cells per dish) (A). Single cells were isolated and plated into 24 well plates (B) and subpopulations were isolated after 2 weeks (C). TSCs were allowed to grow on cover slips to perform immunocytochemistry to investigate the expression of stem cell markers (D). Cell differentiation was induced by culturing TSCs in media without growth factors (bFGF and EGF) and supplemented with serum. Following 30 days of culture in differentiating conditions, TSCs underwent immunocytochemistry for common markers of differentiation (E).
(TIF)

Figure S2 Hypoxia induces PP2A activity independent of HIF-2α expression. (A) TSCs exhibit elevated HIF-2α protein expression 72 hours following exposure to 1% hypoxia, while increased cyclin G2 and hypoxia-induced PP2A activity (bar graph) are seen from 12 hours onwards. Bars represent the mean value of 3 independent experiments ± SEM. (B) Two out of three TSCs express increased HIF-2α protein levels 72 hours following exposure to hypoxia. No detectable HIF-2α is seen in non-stemcell glioblastoma cells (U87) or in TSCs that are differentiated by FBS (10%) containing media without growth factors for 30 days. Cell lysates were also probed with antibodies directed against the A, B and C subunits of PP2A. Hypoxia induces PP2A activity in both TSCs and a non-stemcell tumor cell line (U87). Low PP2A activity is detected in differentiated TSCs. Bars represent the mean value of 3 independent experiments ± SEM.
(TIF)

Figure S3 PP2A mediates reduction of metabolic activity and ATP production during hypoxia. (A) MTT assay reveals partial reversal of hypoxia-mediated growth arrest by PP2A inhibition. Cells are either grown in standard media or in 200 µM CoCl_2_ for 24 hours supplemented with either 0.5 or 1 nM of OA. (B and C) Exposure of hypoxic TSCs to either one or two weeks of 0.1, 0.5 or 1 nM of OA leads to a significant decrease of cell proliferation and viability as determined by MTT. Each bar represents the mean value of 4 independent experiments ± SEM. (D) Measurement of intracellular ATP levels in TSCs in response to hypoxia (1% O_2_). PP2A inhibition significantly delays initial ATP decay seen in hypoxic TSCs within the first 6 to 24 hours. Eventually, PP2A inhibition causes exhaustion of intracellular ATP in hypoxic TSCs. Following 1 and 2 weeks of hypoxia, intracellular ATP levels are significantly decreased in cells treated with OA. Each data point represents the mean value of 6 independent experiments ± SEM. (E) Glucose supplementation partially reverses OA-mediated exhaustion of intracellular ATP levels. TSCs were grown for 1 week in hypoxic conditions (1% O_2_) in high or low glucose media with or without OA followed by measurement of intracellular ATP levels. Each bar represents the mean value of 6 independent experiments ± SEM.
(TIF)

Figure S4 PP2A inhibition enhances death of hypoxic TSCs in a P53-independent fashion (A) Exposure of TSCs to 200 µM of CoCl_2_ induces HIF-1α at all time intervals tested. P53 expression decreases in a time-dependent pattern following incubation with CoCl_2_. Hypoxia is associated with dephosphorylation of P53 at the S15. Bar graph shows the mean phosphorylation coefficient of three independent experiments. (B) TSCs are grown in normal or in CoCl_2_-supplemented culture conditions for six hours. Addition of 0.1 or 1 nM of OA has little effect on P53 expression in normoxic conditions; however, in hypoxic conditions, inhibition of PP2A leads to a dose-dependent recovery of P53 expression. Bar graph depicts mean PP2A activity of 3 independent experiments ± SEM. (C) Western blot demonstrates that shRNA depletes p53 in TSCs. P53 depletion does not alter cell death observed in TSCs cultured in 200 µM CoCl_2_ for one week with concurrent PP2A inhibition.
(TIF)

## References

[1] Stupp R, Mason WP, van den Bent MJ, Weller M, Fisher B (2005). Radiotherapy plus concomitant and adjuvant temozolomide for glioblastoma.. N Engl J Med.

[2] Louis DN, Ohgaki H, Wiestler OD, Cavenee WK, Burger PC (2007). The 2007 WHO classification of tumours of the central nervous system.. Acta Neuropathol.

[3] (2008). Comprehensive genomic characterization defines human glioblastoma genes and core pathways.. Nature.

[4] Libermann TA, Nusbaum HR, Razon N, Kris R, Lax I (1985). Amplification, enhanced expression and possible rearrangement of EGF receptor gene in primary human brain tumours of glial origin.. Nature.

[5] Westermark B, Heldin CH, Nister M (1995). Platelet-derived growth factor in human glioma.. Glia.

[6] Evans SM, Judy KD, Dunphy I, Jenkins WT, Hwang WT (2004). Hypoxia is important in the biology and aggression of human glial brain tumors.. Clin Cancer Res.

[7] Hamada K, Kuratsu J, Saitoh Y, Takeshima H, Nishi T (1996). Expression of tissue factor in glioma.. Noshuyo Byori.

[8] Brat DJ, Castellano-Sanchez AA, Hunter SB, Pecot M, Cohen C (2004). Pseudopalisades in glioblastoma are hypoxic, express extracellular matrix proteases, and are formed by an actively migrating cell population.. Cancer Res.

[9] Amberger-Murphy V (2009). Hypoxia helps glioma to fight therapy.. Curr Cancer Drug Targets.

[10] Zhang TT, Platholi J, Heerdt PM, Hemmings HC, Tung HY (2008). Protein phosphatase-2A is activated in pig brain following cardiac arrest and resuscitation.. Metab Brain Dis.

[11] Yung HW, Tolkovsky AM (2003). Erasure of kinase phosphorylation in astrocytes during oxygen-glucose deprivation is controlled by ATP levels and activation of phosphatases.. J Neurochem.

[12] Heikkinen PT, Nummela M, Leivonen SK, Westermarck J, Hill CS (2009). Hypoxia-activated Smad3-specific dephosphorylation by PP2A.. J Biol Chem.

[13] Cho US, Xu W (2007). Crystal structure of a protein phosphatase 2A heterotrimeric holoenzyme.. Nature.

[14] Janssens V, Goris J (2001). Protein phosphatase 2A: a highly regulated family of serine/threonine phosphatases implicated in cell growth and signalling.. Biochem J.

[15] MacKintosh C, Cohen P (1989). Identification of high levels of type 1 and type 2A protein phosphatases in higher plants.. Biochem J.

[16] Suganuma M, Fujiki H, Suguri H, Yoshizawa S, Hirota M (1988). Okadaic acid: an additional non-phorbol-12-tetradecanoate-13-acetate-type tumor promoter.. Proc Natl Acad Sci U S A.

[17] Fujiki H, Suganuma M (1993). Tumor promotion by inhibitors of protein phosphatases 1 and 2A: the okadaic acid class of compounds.. Adv Cancer Res.

[18] Casillas AM, Amaral K, Chegini-Farahani S, Nel AE (1993). Okadaic acid activates p42 mitogen-activated protein kinase (MAP kinase; ERK-2) in B-lymphocytes but inhibits rather than augments cellular proliferation: contrast with phorbol 12-myristate 13-acetate.. Biochem J.

[19] Puustinen P, Junttila MR, Vanhatupa S, Sablina AA, Hector ME (2009). PME-1 protects extracellular signal-regulated kinase pathway activity from protein phosphatase 2A-mediated inactivation in human malignant glioma.. Cancer Res.

[20] Fishbein JD, Dobrowsky RT, Bielawska A, Garrett S, Hannun YA (1993). Ceramide-mediated growth inhibition and CAPP are conserved in Saccharomyces cerevisiae.. J Biol Chem.

[21] Bennin DA, Don AS, Brake T, McKenzie JL, Rosenbaum H (2002). Cyclin G2 associates with protein phosphatase 2A catalytic and regulatory B′ subunits in active complexes and induces nuclear aberrations and a G1/S phase cell cycle arrest.. J Biol Chem.

[22] Horne MC, Donaldson KL, Goolsby GL, Tran D, Mulheisen M (1997). Cyclin G2 is up-regulated during growth inhibition and B cell antigen receptor-mediated cell cycle arrest.. J Biol Chem.

[23] Ragel BT, Couldwell WT, Gillespie DL, Jensen RL (2007). Identification of hypoxia-induced genes in a malignant glioma cell line (U-251) by cDNA microarray analysis.. Neurosurg Rev.

[24] Bando H, Toi M, Kitada K, Koike M (2003). Genes commonly upregulated by hypoxia in human breast cancer cells MCF-7 and MDA-MB-231.. Biomed Pharmacother.

[25] Leonard MO, Cottell DC, Godson C, Brady HR, Taylor CT (2003). The role of HIF-1 alpha in transcriptional regulation of the proximal tubular epithelial cell response to hypoxia.. J Biol Chem.

[26] Hofstetter CP, Mannaa RH, Mubita L, Anand VK, Kennedy JW (2010). Endoscopic endonasal transsphenoidal surgery for growth hormone-secreting pituitary adenomas.. Neurosurg Focus.

[27] Balss J, Meyer J, Mueller W, Korshunov A, Hartmann C (2008). Analysis of the IDH1 codon 132 mutation in brain tumors.. Acta Neuropathol.

[28] Bleeker FE, Atai NA, Lamba S, Jonker A, Rijkeboer D (2010). The prognostic IDH1(R132) mutation is associated with reduced NADP+-dependent IDH activity in glioblastoma.. Acta Neuropathol.

[29] Pollard SM, Yoshikawa K, Clarke ID, Danovi D, Stricker S (2009). Glioma stem cell lines expanded in adherent culture have tumor-specific phenotypes and are suitable for chemical and genetic screens.. Cell Stem Cell.

[30] Gursel D, Shin BJ, Burkhardt J, Kesavabhotla K, Schlaff CD (2011). Glioblastoma Stem-Like Cells - Biology and Therapeutic Implications.. Cancers.

[31] Gursel DB, Beyene RT, Hofstetter C, Greenfield JP, Souweidane MM (2011). Optimization of glioblastoma multiforme stem cell isolation, transfection, and transduction.. J Neurooncol.

[32] Christensen E (1987). Multivariate survival analysis using Cox's regression model.. Hepatology.

[33] Colella S, Ohgaki H, Ruediger R, Yang F, Nakamura M (2001). Reduced expression of the Aalpha subunit of protein phosphatase 2A in human gliomas in the absence of mutations in the Aalpha and Abeta subunit genes.. Int J Cancer.

[34] Stummer W, Reulen HJ, Meinel T, Pichlmeier U, Schumacher W (2008). Extent of resection and survival in glioblastoma multiforme: identification of and adjustment for bias.. Neurosurgery.

[35] Ivan M, Kondo K, Yang H, Kim W, Valiando J (2001). HIFalpha targeted for VHL-mediated destruction by proline hydroxylation: implications for O2 sensing.. Science.

[36] Jaakkola P, Mole DR, Tian YM, Wilson MI, Gielbert J (2001). Targeting of HIF-alpha to the von Hippel-Lindau ubiquitylation complex by O2-regulated prolyl hydroxylation.. Science.

[37] Lu J, Kovach JS, Johnson F, Chiang J, Hodes R (2009). Inhibition of serine/threonine phosphatase PP2A enhances cancer chemotherapy by blocking DNA damage induced defense mechanisms.. Proc Natl Acad Sci U S A.

[38] Martiniova L, Lu J, Chiang J, Bernardo M, Lonser R (2011). Pharmacologic Modulation of Serine/Threonine Phosphorylation Highly Sensitizes PHEO in a MPC Cell and Mouse Model to Conventional Chemotherapy.. PLoS One.

[39] Korkolopoulou P, Patsouris E, Konstantinidou AE, Pavlopoulos PM, Kavantzas N (2004). Hypoxia-inducible factor 1alpha/vascular endothelial growth factor axis in astrocytomas. Associations with microvessel morphometry, proliferation and prognosis.. Neuropathol Appl Neurobiol.

[40] Heller IH, Elliott KA (1955). The metabolism of normal brain and human gliomas in relation to cell type and density.. Can J Biochem Physiol.

[41] Mahaley MS (1966). The in vitro respiration of normal brain and brain tumors.. Cancer Res.

[42] Warburg O (1926). Über den Stoffwechsel von Tumoren.

[43] Hossmann KA, Mies G, Paschen W, Szabo L, Dolan E (1986). Regional metabolism of experimental brain tumors.. Acta Neuropathol.

[44] Mies G, Paschen W, Ebhardt G, Hossmann KA (1990). Relationship between of blood flow, glucose metabolism, protein synthesis, glucose and ATP content in experimentally-induced glioma (RG1 2.2) of rat brain.. J Neurooncol.

[45] Steinbach JP, Wolburg H, Klumpp A, Probst H, Weller M (2003). Hypoxia-induced cell death in human malignant glioma cells: energy deprivation promotes decoupling of mitochondrial cytochrome c release from caspase processing and necrotic cell death.. Cell Death Differ.

[46] Favaro E, Nardo G, Persano L, Masiero M, Moserle L (2008). Hypoxia inducible factor-1alpha inactivation unveils a link between tumor cell metabolism and hypoxia-induced cell death.. Am J Pathol.

[47] Chowdhury D, Keogh MC, Ishii H, Peterson CL, Buratowski S (2005). gamma-H2AX dephosphorylation by protein phosphatase 2A facilitates DNA double-strand break repair.. Mol Cell.

[48] Goodarzi AA, Jonnalagadda JC, Douglas P, Young D, Ye R (2004). Autophosphorylation of ataxia-telangiectasia mutated is regulated by protein phosphatase 2A.. EMBO J.

[49] Li G, Elder RT, Qin K, Park HU, Liang D (2007). Phosphatase type 2A-dependent and -independent pathways for ATR phosphorylation of Chk1.. J Biol Chem.

[50] Li HH, Cai X, Shouse GP, Piluso LG, Liu X (2007). A specific PP2A regulatory subunit, B56gamma, mediates DNA damage-induced dephosphorylation of p53 at Thr55.. EMBO J.

[51] Yan Y, Cao PT, Greer PM, Nagengast ES, Kolb RH (2010). Protein phosphatase 2A has an essential role in the activation of gamma-irradiation-induced G2/M checkpoint response.. Oncogene.

[52] Jang YJ, Ji JH, Choi YC, Ryu CJ, Ko SY (2007). Regulation of Polo-like kinase 1 by DNA damage in mitosis. Inhibition of mitotic PLK-1 by protein phosphatase 2A.. J Biol Chem.

[53] Wykoff CC, Pugh CW, Maxwell PH, Harris AL, Ratcliffe PJ (2000). Identification of novel hypoxia dependent and independent target genes of the von Hippel-Lindau (VHL) tumour suppressor by mRNA differential expression profiling.. Oncogene.

[54] Mazure NM, Chen EY, Laderoute KR, Giaccia AJ (1997). Induction of vascular endothelial growth factor by hypoxia is modulated by a phosphatidylinositol 3-kinase/Akt signaling pathway in Ha-ras-transformed cells through a hypoxia inducible factor-1 transcriptional element.. Blood.

[55] Chen EY, Mazure NM, Cooper JA, Giaccia AJ (2001). Hypoxia activates a platelet-derived growth factor receptor/phosphatidylinositol 3-kinase/Akt pathway that results in glycogen synthase kinase-3 inactivation.. Cancer Res.

[56] Kawano T, Fukunaga K, Takeuchi Y, Morioka M, Yano S (2001). Neuroprotective effect of sodium orthovanadate on delayed neuronal death after transient forebrain ischemia in gerbil hippocampus.. J Cereb Blood Flow Metab.

[57] Junttila MR, Li SP, Westermarck J (2008). Phosphatase-mediated crosstalk between MAPK signaling pathways in the regulation of cell survival.. FASEB J.

[58] Satoh T, Nakatsuka D, Watanabe Y, Nagata I, Kikuchi H (2000). Neuroprotection by MAPK/ERK kinase inhibition with U0126 against oxidative stress in a mouse neuronal cell line and rat primary cultured cortical neurons.. Neurosci Lett.

[59] Heikkinen PT, Nummela M, Jokilehto T, Grenman R, Kahari VM (2010). Hypoxic conversion of SMAD7 function from an inhibitor into a promoter of cell invasion.. Cancer Res.

[60] Cohen P, Holmes CF, Tsukitani Y (1990). Okadaic acid: a new probe for the study of cellular regulation.. Trends Biochem Sci.

