# Supplementary figures and images for: Protein Phosphatase 2A Mediates Dormancy of Glioblastoma Multiforme-Derived Tumor Stem-Like Cells during Hypoxia
Source: PLoS One. 2012 Jan 11;7(1):e30059. doi: 10.1371/journal.pone.0030059 (PMC3256196; doi:10.1371/journal.pone.0030059)

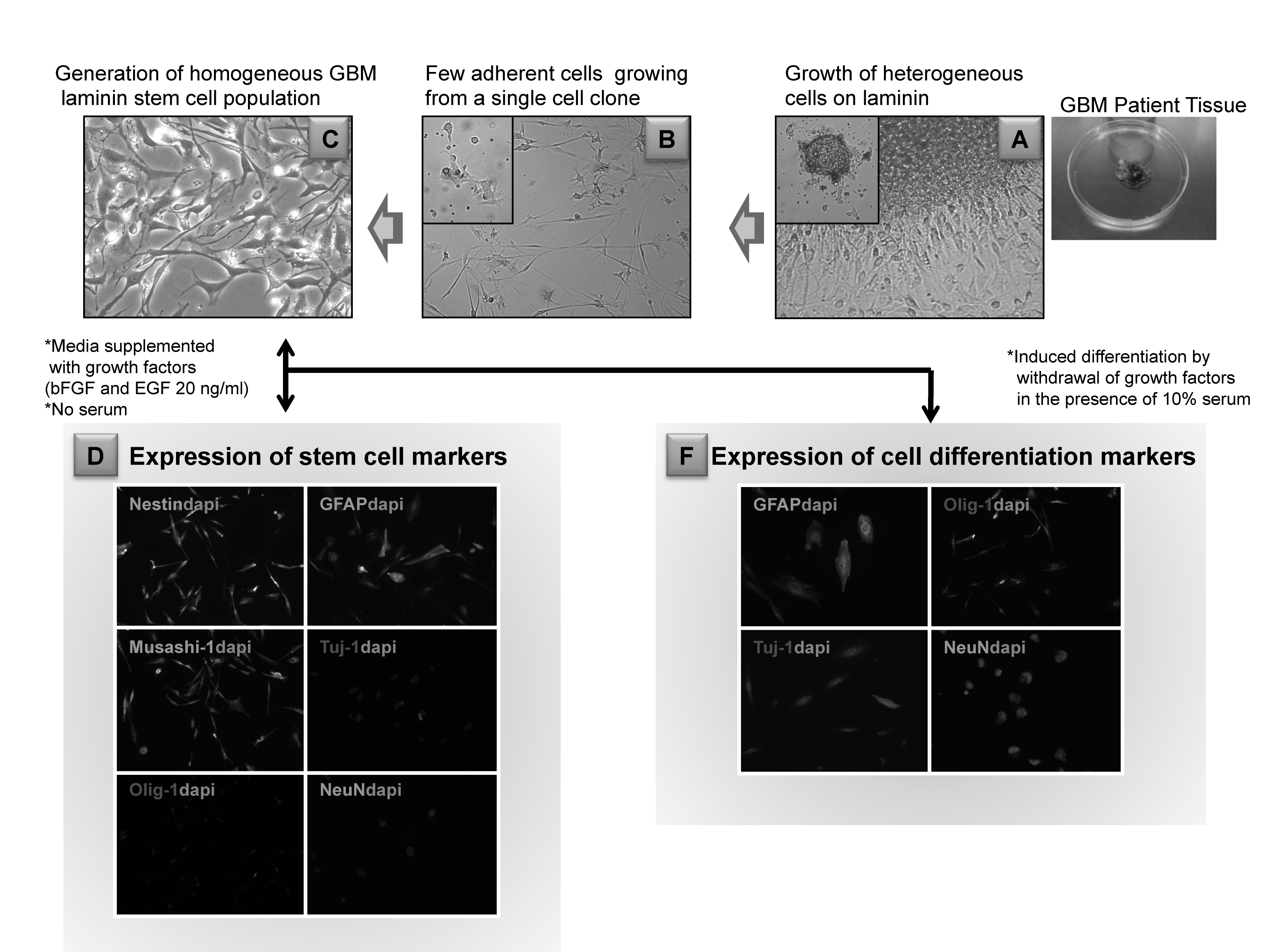

Supplement: Figure S1 — Single cell clonal analysis for stemness and differentiation. Dissociated GBM tissue was seeded on laminin coated 10 cm dishes (3×10^6^ cells per dish) (A). Single cells were isolated and plated into 24 well plates (B) and subpopulations were isolated after 2 weeks (C). TSCs were allowed to grow on cover slips to perform immunocytochemistry to investigate the expression of stem cell markers (D). Cell differentiation was induced by culturing TSCs in media without growth factors (bFGF and EGF) and supplemented with serum. Following 30 days of culture in differentiating conditions, TSCs underwent immunocytochemistry for common markers of differentiation (E). (TIF) [file pone.0030059.s001.tif]

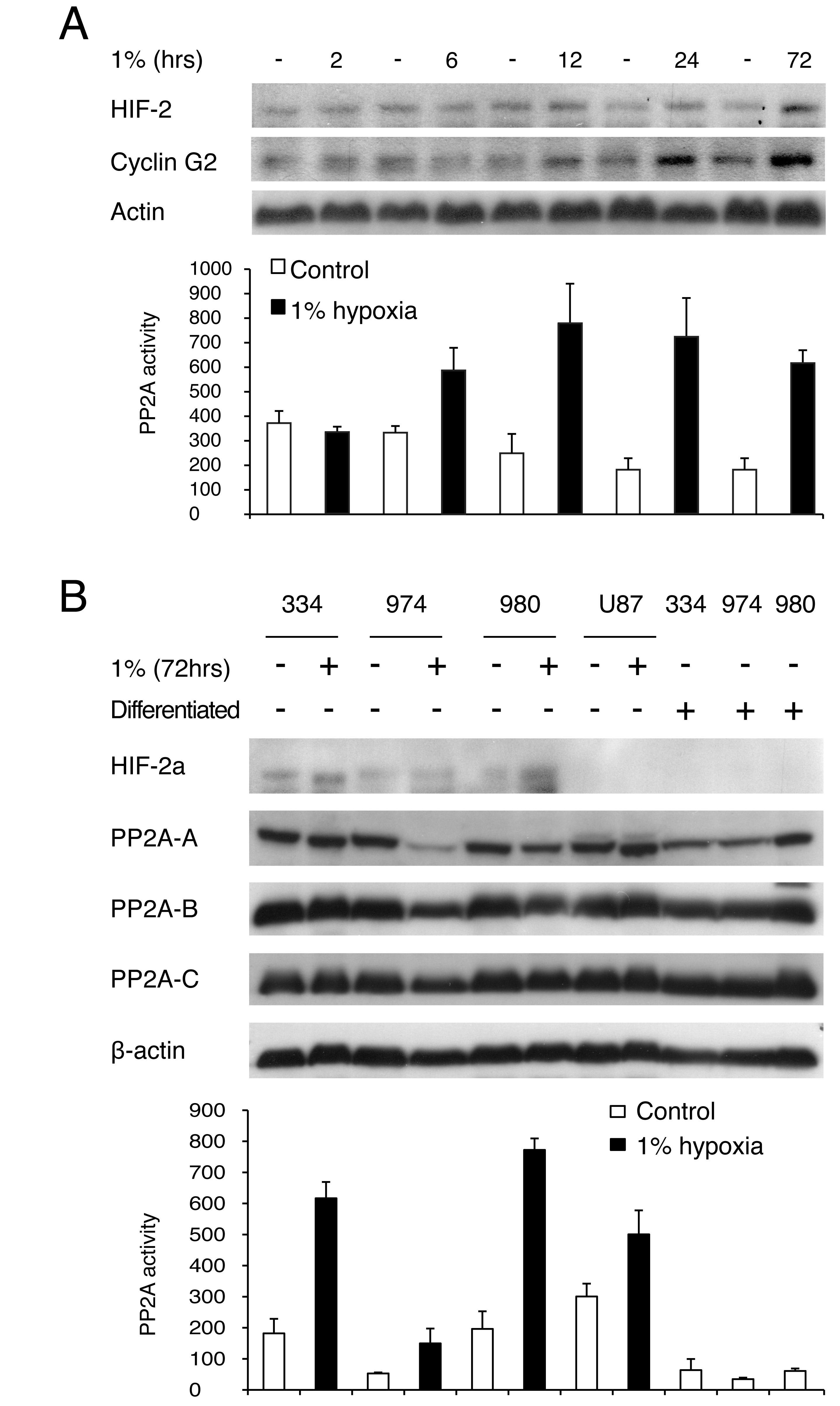

Supplement: Figure S2 — Hypoxia induces PP2A activity independent of HIF-2α expression. (A) TSCs exhibit elevated HIF-2α protein expression 72 hours following exposure to 1% hypoxia, while increased cyclin G2 and hypoxia-induced PP2A activity (bar graph) are seen from 12 hours onwards. Bars represent the mean value of 3 independent experiments ± SEM. (B) Two out of three TSCs express increased HIF-2α protein levels 72 hours following exposure to hypoxia. No detectable HIF-2α is seen in non-stemcell glioblastoma cells (U87) or in TSCs that are differentiated by FBS (10%) containing media without growth factors for 30 days. Cell lysates were also probed with antibodies directed against the A, B and C subunits of PP2A. Hypoxia induces PP2A activity in both TSCs and a non-stemcell tumor cell line (U87). Low PP2A activity is detected in differentiated TSCs. Bars represent the mean value of 3 independent experiments ± SEM. (TIF) [file pone.0030059.s002.tif]

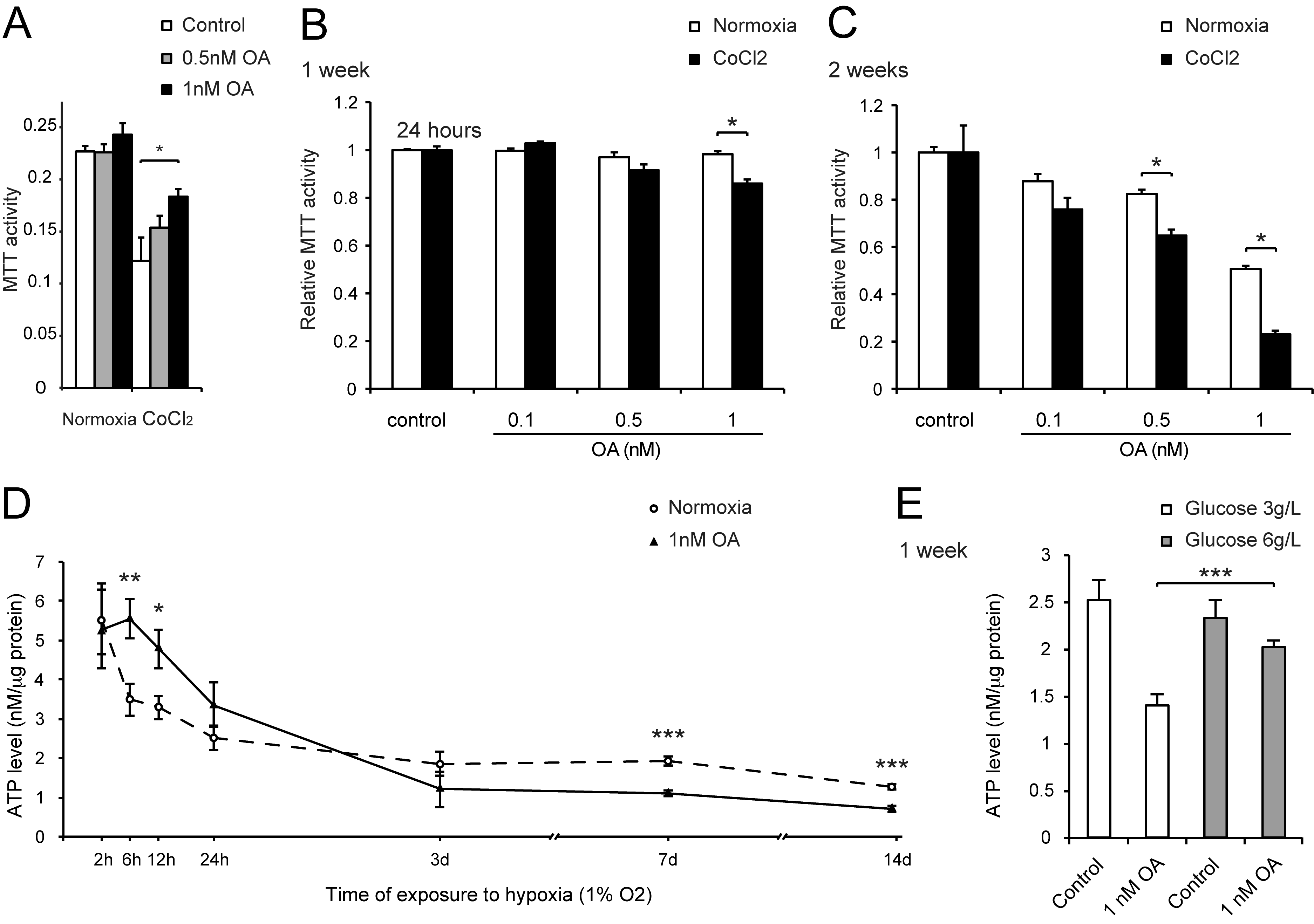

Supplement: Figure S3 — PP2A mediates reduction of metabolic activity and ATP production during hypoxia. (A) MTT assay reveals partial reversal of hypoxia-mediated growth arrest by PP2A inhibition. Cells are either grown in standard media or in 200 µM CoCl2 for 24 hours supplemented with either 0.5 or 1 nM of OA. (B and C) Exposure of hypoxic TSCs to either one or two weeks of 0.1, 0.5 or 1 nM of OA leads to a significant decrease of cell proliferation and viability as determined by MTT. Each bar represents the mean value of 4 independent experiments ± SEM. (D) Measurement of intracellular ATP levels in TSCs in response to hypoxia (1% O2). PP2A inhibition significantly delays initial ATP decay seen in hypoxic TSCs within the first 6 to 24 hours. Eventually, PP2A inhibition causes exhaustion of intracellular ATP in hypoxic TSCs. Following 1 and 2 weeks of hypoxia, intracellular ATP levels are significantly decreased in cells treated with OA. Each data point represents the mean value of 6 independent experiments ± SEM. (E) Glucose supplementation partially reverses OA-mediated exhaustion of intracellular ATP levels. TSCs were grown for 1 week in hypoxic conditions (1% O2) in high or low glucose media with or without OA followed by measurement of intracellular ATP levels. Each bar represents the mean value of 6 independent experiments ± SEM. (TIF) [file pone.0030059.s003.tif]

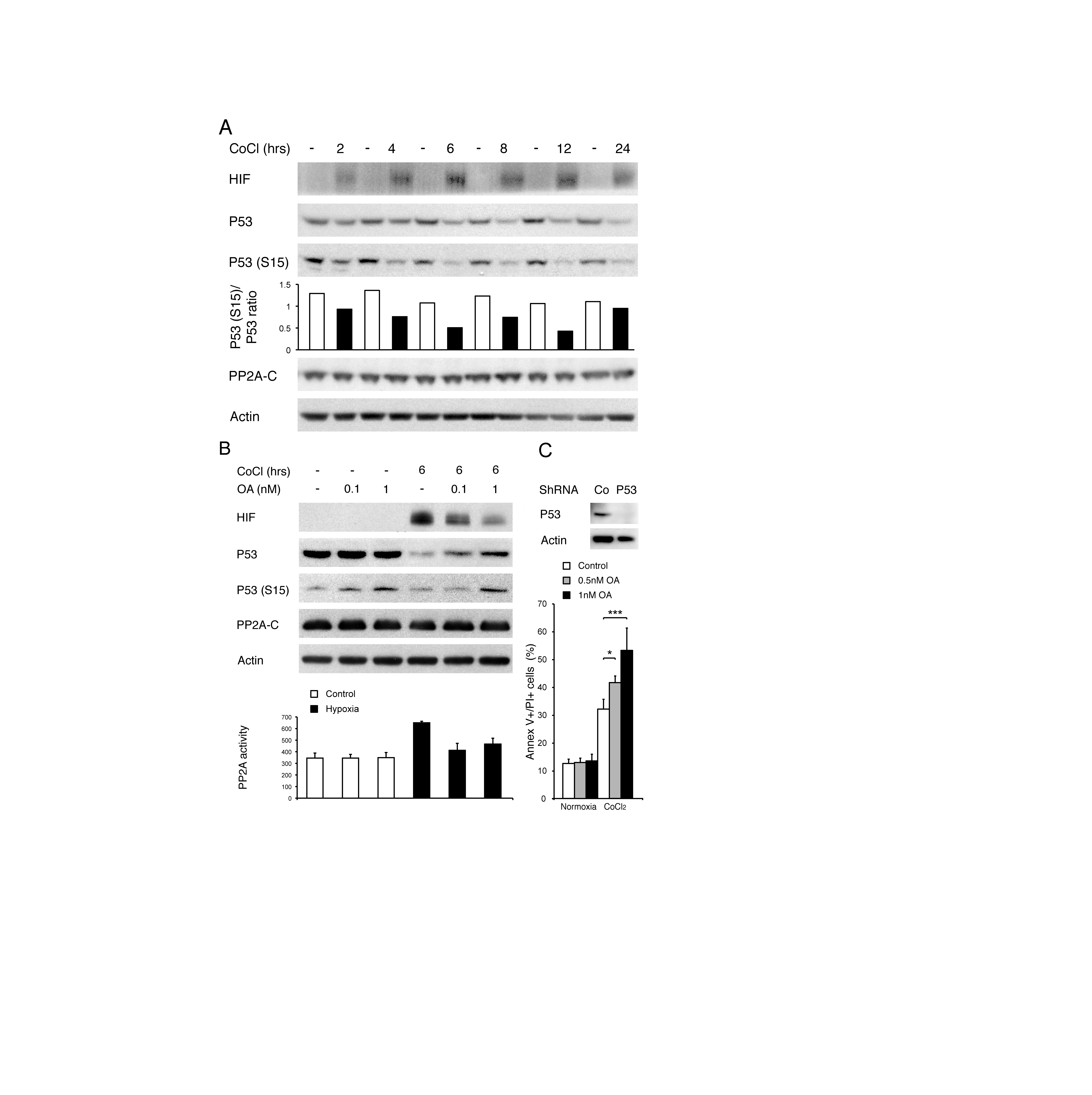

Supplement: Figure S4 — PP2A inhibition enhances death of hypoxic TSCs in a P53-independent fashion (A) Exposure of TSCs to 200 µM of CoCl2 induces HIF-1α at all time intervals tested. P53 expression decreases in a time-dependent pattern following incubation with CoCl2. Hypoxia is associated with dephosphorylation of P53 at the S15. Bar graph shows the mean phosphorylation coefficient of three independent experiments. (B) TSCs are grown in normal or in CoCl2-supplemented culture conditions for six hours. Addition of 0.1 or 1 nM of OA has little effect on P53 expression in normoxic conditions; however, in hypoxic conditions, inhibition of PP2A leads to a dose-dependent recovery of P53 expression. Bar graph depicts mean PP2A activity of 3 independent experiments ± SEM. (C) Western blot demonstrates that shRNA depletes p53 in TSCs. P53 depletion does not alter cell death observed in TSCs cultured in 200 µM CoCl2 for one week with concurrent PP2A inhibition. (TIF) [file pone.0030059.s004.tif]
